# Node Non-Uniform Deployment Based on Clustering Algorithm for Underwater Sensor Networks

**DOI:** 10.3390/s151229786

**Published:** 2015-12-01

**Authors:** Peng Jiang, Jun Liu, Feng Wu

**Affiliations:** 1 Key Lab for IOT and Information Fusion Technology of Zhejiang, Hangzhou 310018, China; liujunhdu@163.com (J.L.); fengwu@hdu.edu.cn (F.W.); 2 College of Automation, Hangzhou Dianzi University, Hangzhou 310018, China

**Keywords:** underwater sensor networks, non-uniform deployment, clustering, network lifetime

## Abstract

A node non-uniform deployment based on clustering algorithm for underwater sensor networks (UWSNs) is proposed in this study. This algorithm is proposed because optimizing network connectivity rate and network lifetime is difficult for the existing node non-uniform deployment algorithms under the premise of improving the network coverage rate for UWSNs. A high network connectivity rate is achieved by determining the heterogeneous communication ranges of nodes during node clustering. Moreover, the concept of aggregate contribution degree is defined, and the nodes with lower aggregate contribution degrees are used to substitute the dying nodes to decrease the total movement distance of nodes and prolong the network lifetime. Simulation results show that the proposed algorithm can achieve a better network coverage rate and network connectivity rate, as well as decrease the total movement distance of nodes and prolong the network lifetime.

## 1. Introduction and Related Works

Underwater sensor networks (UWSNs) are an underwater monitoring system comprised of nodes that are capable of sensing underwater information, processing information data and communicating through underwater acoustic signal. Compared with the traditional terrestrial wireless sensor networks (WSNs), the UWSNs monitoring system owns different characteristics. First, the acoustic signal is usually chosen as the communication medium, since the optical or radio-frequency signal attenuates very rapidly in underwater environment. Second, in UWSNs, nodes are usually deployed in a 3-D environment, which introduces new challenges in terms of connectivity, coverage and mobility. Third, since battery re-charging hundreds of meters below the water surface is difficult and expensive, the energy efficiency in UWSNs seems to deserve more consideration [1,2]. The problem of the node non-uniform deployment for UWSNs means that under the premise of fully considering the above characteristics, the nodes must be deployed non-uniformly according to the distribution of the cover targets, *i.e.*, the events, whose distribution in the monitored water space may be non-uniform. Meanwhile, the deployment of nodes should increase the network coverage rate for the events and the network connectivity rate, and should decrease the energy consumption, as well as prolong the lifetime of the network.

On the one hand, several studies have recently investigated the node non-uniform deployment for UWSNs [3]. Considering the specific application of monitoring water quality in a small closed area (e.g., a lake), Aitsaadi *et al.* [4] proposed differentiated deployment algorithm based on a mesh representation method inspired from the image processing and 3-D modeling field. Sensors were deployed non-uniformly according to the distribution of the pollutants. The algorithm can achieve a good coverage of the pollutants but cannot attain a high network connectivity rate. Golen *et al.* [5] determined the possibilities of the events’ appearances in the monitored water space sub-regions with the help of solving the minimax game matrix. Accordingly, the number of nodes that must be allocated in each sub-region were calculated. The algorithm could also achieve good coverage but was not related to the specific solution on how the nodes must be deployed. Moreover, the algorithm could not achieve a high network connectivity rate. Xia *et al.* [6] initially proposed the fish-inspired node non-uniform deployment algorithm for UWSNs and subsequently proposed the similar particle swarm-inspired node deployment (PSIND) algorithm [7]. By simulating the fish or particle behavior and introducing a crowd control, the proposed algorithms can drive nodes to cover the events and to be distributed to match the distribution of the events. However, the authors only considered the network coverage rate and did not consider the network connectivity rate. In addition, these algorithms had a number of nodes move, and nodes may move blindly during the deployment. Given the limited energy of the node and its difficulty to be recharged, as well as the large movement energy consumption in the water, these defects may cause premature deaths of nodes because of energy exhaustion, which shortens the network lifetime. On the other hand, many studies [8,9,10,11] have also investigated network clustering in WSNs. Abbasi *et al.* [12] surveyed different clustering algorithms for the WSNs, highlighting their objects, features, complexities, *etc.* Additionally, the authors claimed that network clustering can improve the network connectivity as well as prolong the network lifetime. Lloret *et al.* [13] proposed an algorithm that can structure the topology of different WSNs to coexist in the same environment based on network clustering, where cluster head nodes manage their own networks and have connections with other cluster head nodes, resulting in good network connectivity and scalability of the whole parallel network structure. Tsai *et al.* [14] considered the energy conservation benefits of hierarchical protocols as well as data reduction, and the authors proposed a sub-clustering procedure based on spatial data correlation to further separate the hierarchical (clustered) architecture of the WSNs. The procedure can effectively conserve energy and monitor accurately the environment within an acceptable level.

Optimization of the network connectivity rate and network lifetime is difficult for the existing node non-uniform deployment algorithms under the premise of improving the network coverage rate for UWSNs. Thus, a node non-uniform deployment based on a clustering (NNDBC) algorithm for UWSNs is proposed. The heterogeneous communication ranges of nodes are determined during node clustering, which promotes the network connectivity rate. Moreover, the concept of aggregate contribution degree is defined, and the nodes with lower aggregate contribution degrees are used to substitute the dying nodes because this can help decrease the number of nodes moving during the deployment and avoid the blind movement of nodes. Thus, the total movement distance of nodes is decreased, and the network lifetime is prolonged. Simulation results show that compared with the typical node non-uniform deployment PSIND algorithm, the NNDBC algorithm can achieve a better network coverage rate and a better network connectivity rate during network operation, as well as decrease the total movement distance of nodes and prolong the network lifetime.

Compared with the existing related algorithms, the contribution of our work is as follows:

(1) Based on the clustering method, the heterogeneous communication ranges of nodes are a preferred approach to promote the network connectivity rate, rather than the homogeneous communication ranges of nodes.
(2) The concept of aggregate contribution degree is defined, and the nodes with lower aggregate contribution degrees are used to substitute the dying nodes because this can help decrease the number of moving nodes during the deployment and avoid the blind movement of nodes. Consequently, the total movement distance of nodes is decreased, and the network lifetime is prolonged.

The rest of this paper is organized as follows: in Section 2, the preliminaries, models and definitions related to the NNDBC algorithm are formally introduced. In Section 3, we elaborate the problem of the node non-uniform deployment for UWSNs and the corresponding NNDBC algorithm. The simulation evaluation is provided in Section 4 with our conclusions in Section 5.

## 2. Preliminaries, Models and Definitions

### 2.1. Preliminaries

(1) The monitored 3-D underwater space is modelled as a large cube, where the events to be detected are distributed randomly. The number of events is *e_t_*.
(2) All the nodes can locate themselves with the help of related localization algorithms, such as the hierarchical localization algorithm [15].
(3) All the nodes have the ability to move freely in all directions with the help of related technology, such as autonomous underwater vehicles technology [16]. The moving speed of nodes is sufficiently fast; thus, the moving time of nodes can be neglected.
(4) All the nodes have the same initial energy *E_ini_* and the sensing range *R_s_*. The communication ranges of nodes vary from low to high levels, and different levels indicate different communication ranges. A node can adjust its communication level if necessary.
(5) The information packages transmitted among the nodes can be classified into two types, namely, the event and topology information packages. The former information packages are used to store event information. The latter information packages are used to store information that can help form, maintain and optimize the network topology. The size of the event and topology information packages is *M*.

### 2.2. Models

#### 2.2.1. Network Clustering Model

**Figure 1:**
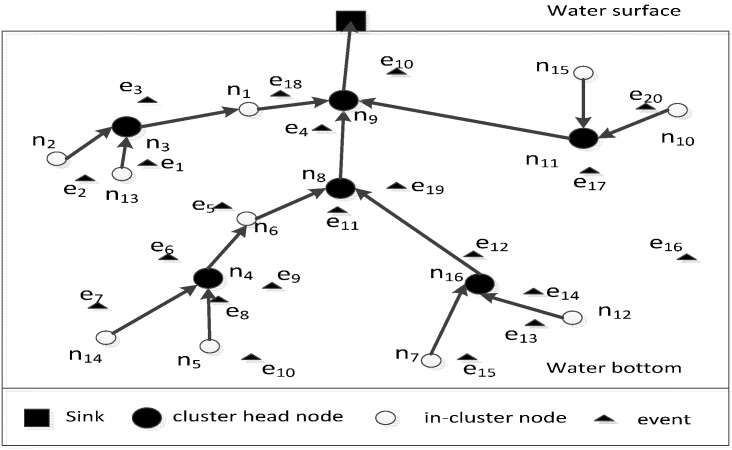
Network clustering model.

Figure 1 shows the clustering of UWSNs searched in this paper. For example, there are 16 nodes and 20 events in the underwater space. After the nodes are distributed randomly in the underwater space, the clustering method described in this paper is used to form the cluster structure of UWSNs. The nodes in a cluster consist of in-cluster nodes and cluster head node. The in-cluster nodes are connected to its own cluster head node, whereas the cluster head nodes are connected to a closest node which is in one of other clusters and is in a shallower location. In this way, the entire network can achieve full connectivity.

#### 2.2.2. Node Energy Consumption Model

Considering that the energy consumption of nodes for sensing, processing and receiving information is much smaller than for transmitting information and moving [17], only the latter is considered. The energy consumption for transmitting information is modeled based on the method mentioned in [18,19]. Supposing that *P_r_* denotes the power threshold for a node to receive the information package, and *d* denotes the transmitting distance of the information package, the energy consumption for transmitting information is denoted as *E_tx_(d)*, which can be calculated using the following formula:

(1) $$E_{tx}\left(d\right)=P_{r}\times T_{p}\times A\left(d\right)$$

where *T_p_* denotes the transmitting time of the information package and can be calculated as follows:

(2) $$T_{p}=\frac{M_{b}}{S_{v}}$$

where *M_b_* is the size of the information package, and *S_v_* is the transmission speed of the information package. *A(d)* denotes energy attenuation when the transmitting distance of the information package is *d* and can be calculated as follows:

(3) $$A\left(d\right)=d^{\lambda }\times \beta^{d}$$

where λ is the energy spreading factor (λ is 1 for cylindrical, 1.5 for practical and 2 for spherical spreading). The parameter $\beta =10^{\alpha \left(f\right)/10}$ is determined by the absorption coefficient α*(f)*, which can be calculated using the following equation:

(4) $$\alpha \left(f\right)=0.11\frac{10^{-3}f^{2}}{1+f^{2}}+44\frac{10^{-3}f^{2}}{4100+f^{2}}+2.75\times 10^{-7}f^{2}+3\times 10^{-6}$$

where *f* is the frequency of the carrier acoustic signal in KHZ, and α*(f)* is in dB/m. The movement energy consumption *M_e_* can be defined as the product of the movement distance *m_d_* and the energy consumption per movement distance *m_u_*, which can be also described as follows:

(5) $$M_{e}=m_{d}*m_{u}$$

#### 2.2.3. Varying Communication Level Model for Maximum Communication Range

Supposing that the maximum communication range is denoted as *R_c_* and is initialed to be *R_ini_*. The corresponding communication level for the maximum communication range is denoted as *L_ev_* and is initialed to be 1. The relationship between the maximum communication range and the corresponding communication level is as follows:

(6) $$R_{c}=R_{ini}+(L_{ev}-1)\times B_{u}$$

where *B_u_* is the increasing communication range per communication level.

### 2.3. Definitions

#### 2.3.1. Network Coverage Rate

The cover targets in this study are the isolated events in the monitored underwater space, and the network coverage rate can be defined as the following Equation (8):

(7) $$C_{or}=\frac{e_{c}}{e_{t}}$$

where *e_c_* is the number of events covered by nodes, and *e_t_* is the total number of events distributed in the monitored underwater space.

#### 2.3.2. Network Connectivity Rate

The network connectivity rate *C_n_* can be defined as the ratio of *n_c_* and *n*, where *n_c_* is the number of nodes that can communicate with the sink node through single-hop or multi-hop communication [20]. *C_n_* can be calculated as follows:

(8) $$C_{n}=\frac{n_{c}}{n}$$

If the network connectivity rate is 1, the network achieves full network connectivity, and all the nodes can communicate with the sink node through single-hop or multi-hop communication.

#### 2.3.3. Backbone Node

During the transmission of information packages from nodes to the sink node, if a node helps transmit the information packages, it can be defined as a backbone node, which can be denoted as *N_g_*. The backbone nodes include all the cluster head nodes and several special in-cluster nodes. As shown in Figure 1, the backbone nodes include all the cluster head nodes (*i.e.*, *n*_3_, *n*_4_, *n*_8_, *n*_9_, *n*_11_, *n*_16_) and several special in-cluster nodes (*i.e.*, *n*_1_, *n*_6_).

#### 2.3.4. Aggregate Contribution Degree

1. Coverage Contribution Degree

The coverage contribution degree of a node is denoted as *C_vd_*, which indicates the number of events changing from the covered to the uncovered state when the node turns off its sensing module. Figure 1 shows that in a cluster that consists of nodes *n*_7_, *n*_12_ and *n*_16_. The event *e*_15_ is covered by the node *n*_7_, and the events *e*_13_ and *e*_14_ are covered by the node *n*_12_. Meanwhile, the events *e*_12_ and *e*_14_ are covered by the node *n*_16_. Although the nodes *n*_12_ and *n*_16_ cover two events, the event *e*_12_ is covered by both of them; thus, the coverage contribution degrees of the nodes *n*_12_ and *n*_16_ are the same, which is 1.

2. Connectivity Contribution Degree

The connectivity contribution degree of a node is denoted as *C_nd_*. Regarding the backbone node, its connectivity contribution degree indicates the number of backbone nodes which use itself as the relay node to transmit the information messages to the sink node. Figure 1 shows that the connectivity contribution degrees of the backbone nodes *n*_3_, *n*_4_, *n*_11_ and *n*_16_ are 0, whereas those of the backbone nodes *n*_1_ and *n*_6_ are 1, and those of the backbone nodes *n*_8_ and *n*_9_ are 3 and 7 respectively. As to the non-backbone node, its connectivity contribution degree is 0.

3. Aggregate Contribution Degree

The aggregate contribution degree of a node is denoted as *C_md_*, which indicates the weighted sum of the coverage contribution degree and the connectivity contribution degree, which can be shown as Equation (9):

(9) $$C_{md}=a\;\times \;C_{nd}+\;b\;\times \;C_{vd}$$

where *a* and *b* are the weighted coefficients. The value of *a* must be larger than that of *b* to ensure that the NNDBC algorithm can achieve a high network connectivity rate.

#### 2.3.5. Network Lifetime

The network lifetime is denoted as *L_i_*, which is one of the important criteria to evaluate the energy efficiency of the algorithms [21,22]. In this paper, the network lifetime is defined as the operating rounds where the network coverage rate *C_or_* satisfies the condition (*i.e.*, $C_{th}\leq C_{or}\leq 100\%$), and *C_th_* is the coverage rate threshold. If the network coverage rate is smaller than *C_th_*, the network has difficulty in monitoring the underwater space, and the network lifetime is over.

#### 2.3.6. Equivalence Maximum Communication Range

In the NNDBC algorithm, the communication ranges of nodes are heterogeneous. For different nodes or different operating rounds of the same node, the communication ranges may be different. In the PSIND algorithm, the communication ranges of nodes are homogeneous, which means the communication ranges of all nodes are the same and remain unchanged during the entire network operation. To provide some convenience for the simulation and related evaluation, we define the equivalence maximum communication range *R_e_* for the NNDBC algorithm, and it’s shown as the following Equation (10):

(10) $$R_{e}=\frac{\sum_{i=1}^{n}R_{a}(i)}{n}$$

where *n* is the total number of nodes and *R_a_(i)* is the average maximum communication range of node *i* during the network operation, which can be shown as the following Equation (11):

(11) $$R_{a}(i)=\frac{\sum_{z=1}^{V_{i}}R_{z}}{V_{i}}$$

where *V_i_* is the life rounds of node *i*, and *R_z_* denotes the maximum communication range of node *i* at the round *z*.

#### 2.3.7. Reconstruction Node Rate

After nodes are randomly distributed in the monitored underwater space, if any node moves again during the network operation, we define that this node takes part in the network reconstruction. The reconstruction rate *C_gr_* can be defined as the ratio between the number of nodes taking part in the network reconstruction (*i.e.*, *n_g_*) and the total number of nodes (*i.e.*, *n*), which can be shown as the following Equation (12):

(12) $$C_{\text{gr}}=\frac{n_{g}}{n}$$

## 3. Description of Problem and Algorithm

### 3.1. Problem Description

As to the problem of the node non-uniform deployment for UWSNs, the cover targets are the events whose distribution in the monitored underwater space is non-uniform. The deployment algorithm must ensure the network coverage rate on the events. It must also improve the network connectivity rate as high as possible and decrease the energy consumption of nodes during the deployment, which prolongs the network lifetime. However, existing non-uniform deployment algorithms for UWSNs usually only consider how to improve the network coverage rate and ignore how to optimize the network connectivity rate and the network lifetime. For example, Xia *et al.* [6] initially proposed the fish-inspired node non-uniform deployment algorithm for UWSNs. Basing on this algorithm, they proposed the similar PSIND algorithm [7]. By simulating the fish or particle behavior and introducing a crowd control, the proposed algorithm can drive the nodes to cover the events and to be distributed to match the distribution of the events. However, the authors only considered the network coverage rate and did not consider the network connectivity rate. These algorithms also had a number of nodes move, and nodes may move blindly during the deployment. Given the limited energy of the node and its difficulty to be recharged, as well as the large movement energy consumption in the water, these defects may cause premature deaths of nodes because of energy exhaustion, which shortens the network lifetime.

To offer a better solution for the problem of the node non-uniform deployment for UWSNs, we propose a NNDBC algorithm for UWSNs. The heterogeneous communication ranges of nodes are determined during node clustering, which promotes the network connectivity rate. Moreover, the concept of aggregate contribution degree is defined, and the nodes with lower aggregate contribution degrees are used to substitute the dying nodes because this can help decrease the number of moving nodes during the deployment and avoid the blind movement of nodes. The total movement distance of nodes is decreased, and the network lifetime is prolonged.

### 3.2. Algorithm Description

The NNDBC algorithm consists of two phases, namely, the initial adjustment and steady operating phases. The first phase is as follows:

(1) In the monitored underwater space whose volume is *V*, the sink node is located at the center of the water surface, and the number of events distributed in the space is *e_t_*. All the nodes adjust their depths randomly to cover as many events as possible after they are scattered (usually scattered by the plane or the ship) on the surface of the underwater space.
(2) The communication levels, *i.e.*, *L_ev_* of all the nodes, are initialed to be 1. All the nodes broadcast the neighbor finding messages *M_f_* and attempt to receive that from others. If node *i* receives the neighbor finding messages *M_f_* from node *j*, node *i* changes its communication level to be the same with that of node *j*, and then node *i* replies the response message *M_a_* to node *j*. If node *i* cannot receive any neighbor finding message *M_f_*, node *i* is in an isolated state; thus, it gradually increases its communication level one by one while broadcasts its neighbor finding messages *M_f_*. The above process continues until all the nodes receive the response message *M_a_*, that is, there is no isolated node in the network.
(3) All the nodes detect their surrounding events and then, broadcast the cluster forming messages *C_s_*, which includes the information of the number of the events detected. Given that the backbone nodes, which consist of cluster head nodes and several special in-cluster nodes, are prioritized over the non-backbone nodes to be substituted when they are dying to improve the network coverage rate, node *i* chooses the node that covers the most events from its neighboring nodes (including itself) to be its cluster head node. If more than one neighboring node covers the same maximum number of events, node *i* chooses the node which is closest to itself as its cluster head node. Each in-cluster node and cluster head node adjusts its communication level to communicate with its cluster head node and in-cluster node, respectively. Each cluster in the network gets internally connected.
(4) After step (3), if the possible isolated cluster is eliminated, the entire network can be evidently connected and achieve full network connectivity. Thus, each cluster head node chooses the closest node which is in other clusters (including the cluster head node and in-cluster node) and is in a shallower (*i.e.*, with the smaller depth) location to be its next-hop during the transmission of messages to the sink node. Each in-cluster node chooses its cluster head node to be its next-hop. Eventually, all the nodes that act as message transmitting nodes form a connected backbone, and the network gets fully connected, and the initial adjustment phase ends.

**Figure 2:**
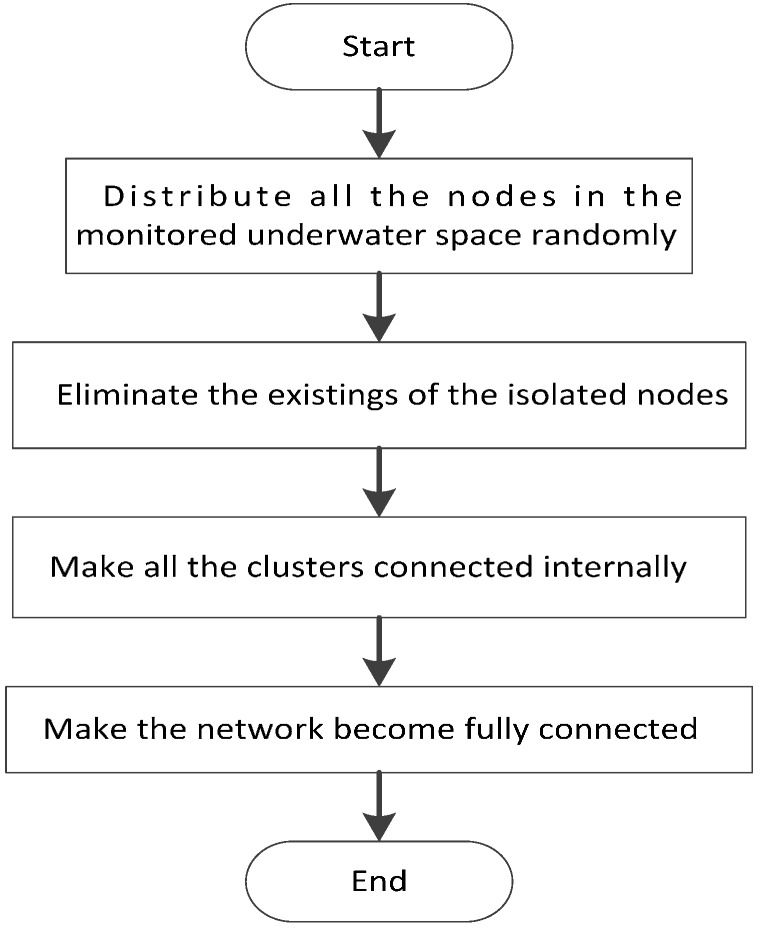
Initial adjustment phase of NNDBC algorithm.

This entire phase is shown in Figure 2.

After the initial adjustment phase, the network gets fully connected, and the second phase (*i.e.*, steady operating phase) begins. The network operates round by round until the network lifetime when the network coverage rate *C_n_* is smaller than the coverage rate threshold *C_th_*. In every round, each node detects its surrounding events and transmits the information detected to the sink node through single-hop or multi-hop. During the network operation, if node *i* is dying (*i.e.*, its left energy is smaller than the energy threshold *E_th_*), it must be substituted by other nodes in the following way:

(1) First, the cluster head node *C(i)* of node *i* determines whether any live node whose aggregate contribution degree is smaller than that of node *i* in its cluster exists. If it exists, the cluster head node *C(i)* regulates the one that possesses the smallest aggregate contribution degree to help node *i*, requiring that node to move along the straight line to substitute node *i*. If the node is absent, then the cluster head node *C(i)* broadcasts the help-need message *H_m_* to other cluster heads through single-hop or multi-hop.
(2) The cluster head node that has received the help-need message *H_m_* determines whether any live node whose aggregate contribution degree is smaller than that of node *i* in its cluster exists. If the node exists, the cluster head node responds to the cluster head node *C(i)* with the help-give message *G_m_*, revealing the nodes that can offer help to *C(i)*. If the cluster head node *C(i)* can receive the help-give message *G_m_* (which may be more than one), it chooses the closest node *h* that can offer help and transmits the request message *R_m_* to the cluster head node *C(h)* of node *h*. After receiving the request message *R_m_*, the cluster head node *C(h)* requires the node *h* to move along the straight line to substitute node *i*. If the cluster head node *C(i)* cannot receive any *G_m_*, the node *i* cannot be substituted by any node.

Given that the aggregate contribution degrees of the backbone nodes are usually larger than those of the non-backbone nodes during network operation, the nodes substituted are usually the backbone nodes, and the nodes offering help are usually the non-backbone nodes.

## 4. Simulation Evaluation

### 4.1. Algorithm Comparison and Evaluation Metrics

PSIND is a typical node non-uniform deployment algorithm for UWSNs; thus, this algorithm is chosen for comparison. The performances of PSIND and NNDBC algorithms from the following evaluation metrics are compared: the network coverage rate, network connectivity rate, reconstruction node rate and network lifetime.

### 4.2. Simulation Scenario and Parameter Settings

Matlab software is used to simulate the algorithms. The final results shown in the following figures are the averages of the 30 simulation times to eliminate the effect of the simulation randomness. The length, width and depth of the monitored simulative 3-D water space are the same (100 m). The sink node is located at the center of the monitored 3-D water space, where events (the number of which (*i.e.*, *e_t_*) is 100) are located. Several parameters of the NNDBC algorithm are set as follows: initial maximum communication range, *R_ini_* = 20 m and increasing communication range per communication level *B_u_* = 5 m. The weighted coefficients *a* and *b* in calculating the aggregate contribution degree are 50 and 1, respectively. Some of the parameters of the PSIND algorithm are set as follows: the expecting cover level of the event (*i.e.*, *D*) is 1, and the maximum distance in each movement of a node (*i.e.*, *l_max_*) is 15 m, and the maximum communication range *R_c_* = *Re*, where *Re* is the equivalence maximum communication range of the NNDBC algorithm. The operation cycle of the PSIND algorithm is 10 rounds where the network topology is adjusted according to the PSIND algorithm every 10 rounds. Other common parameters are shown in Table 1.

**Table 1:** Parameter settings.

Parameter Names	Parameter Values
Initial energy of node (*E_i_*)	1000 J
Node energy threshold (*E_th_*)	10 J
Network coverage rate threshold (*C_th_*)	0.1
Energy consumption per movement distance (*m_u_*)	1.5 J/m
Size of information package (*M_b_*)	1 Kbit
Power threshold (*P_r_*)	0.05 w
Frequency of carrier acoustic signal (*f*)	25 kHz
Transmission speed of information package (*S_v_*)	5 kbps
Energy spreading factor (λ)	1.5
Sensing range of node (*R_s_*)	15 m

### 4.3. Simulation Results and Analyze

**Figure 3:**
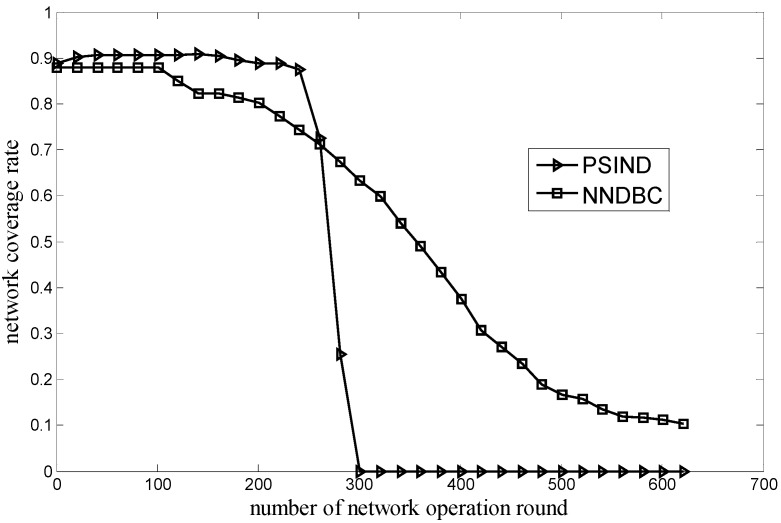
Comparison of network coverage rate during network operation.

Figure 3 shows the comparison of the relationship between the network coverage rate and the round of network operation for the NNDBC and PSIND algorithms, where the number of nodes and events of the simulated scenario are 30 and 100, respectively. As shown in Figure 3, if the round of network operation is the same with that of the NNDBC algorithm, the PSIND algorithm can obtain a higher network coverage rate in a long period of rounds (*i.e.*, from the beginning to the round 261) during its network lifetime (*i.e.*, round 292). This phenomenon is attributed to the reason that the PSIND algorithm only focuses on how to improve the network coverage rate, attempting to drive the nodes to the areas where more events are distributed. However, the network lifetime of the PSIND algorithm is much smaller than that of the NNDBC algorithm (*i.e.*, round 627). Compared with the PSIND algorithm, the NNDBC algorithm can obtain a relatively high network coverage rate for longer operation time. The reason is that the NNDBC algorithm can decrease the energy consumption of nodes during the network topology adjustment by decreasing node movement; therefore, nodes can reserve more energy to sustain longer detecting time and obtain a better network coverage rate.

**Figure 4:**
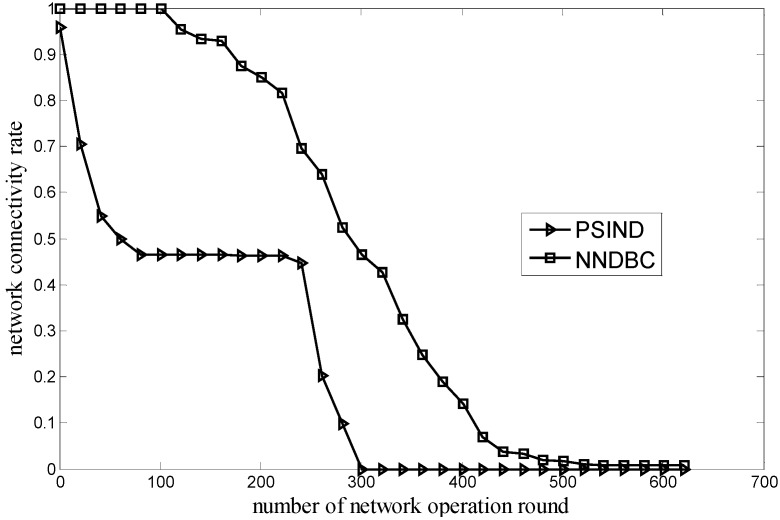
Comparison of network connectivity rate during network operation.

Figure 4 shows the comparison of the relationship between the network connectivity rate and the round of network operation for the NNDBC and PSIND algorithms, where the number of nodes and events of the simulated scenario are 30 and 100, respectively. As shown in Figure 4, if the round of network operation is the same with that of the PSIND algorithm, the NNDBC algorithm can obtain a higher network connectivity rate. Moreover, for the NNDBC algorithm, the network can achieve a completely full network connectivity in the starting rounds (*i.e.*, form the beginning to round 100) and maintain a relatively high network connectivity rate for longer operation time. The reason is that for the NNDBC algorithm, the heterogeneous communication ranges of nodes are determined during node clustering. The formation of the backbone helps improve the network connectivity rate, making the network achieve full network connectivity rate at the starting rounds of the network operation. During the network operation, if the backbone node is dying, it can be substituted by the non-backbone node. This is also helpful to improve the network connectivity rate.

**Figure 5:**
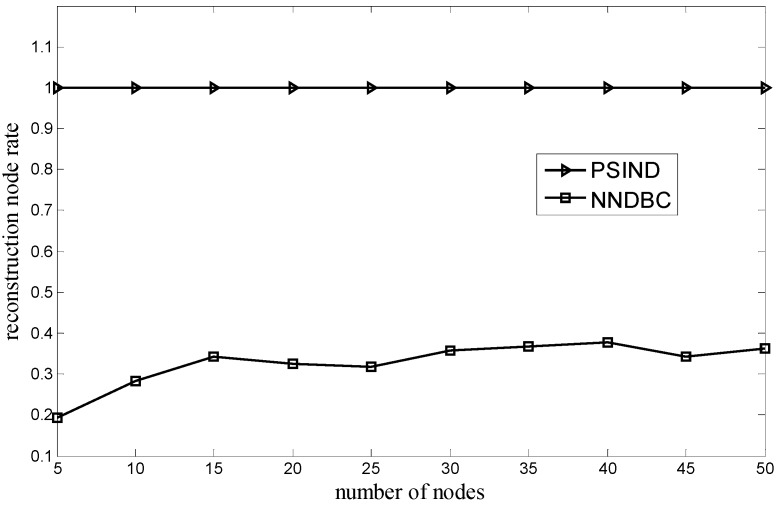
Comparison of reconstruction node rate when number of nodes varies.

Figure 5 shows the comparison of the relationship between the reconstruction node rate and the number of nodes for the NNDBC and PSIND algorithms, where the number of nodes and the number of events of the simulated scenario are varied and 100, respectively. As shown in Figure 5, the reconstruction node rate for the PSIND algorithm is always 1, and if the number of nodes is the same with that of the PSIND algorithm, the NNDBC algorithm can obtain a lower reconstruction node rate. The reason is that the PSIND algorithm ignores the optimization on the node movement during the network operation, and all the nodes have to move to participate in the network topology adjustment. However, the NNDBC algorithm only requires the nodes offering help to move to the locations of the nodes that need to be substituted, thus decreasing the reconstruction node rate dramatically.

**Figure 6:**
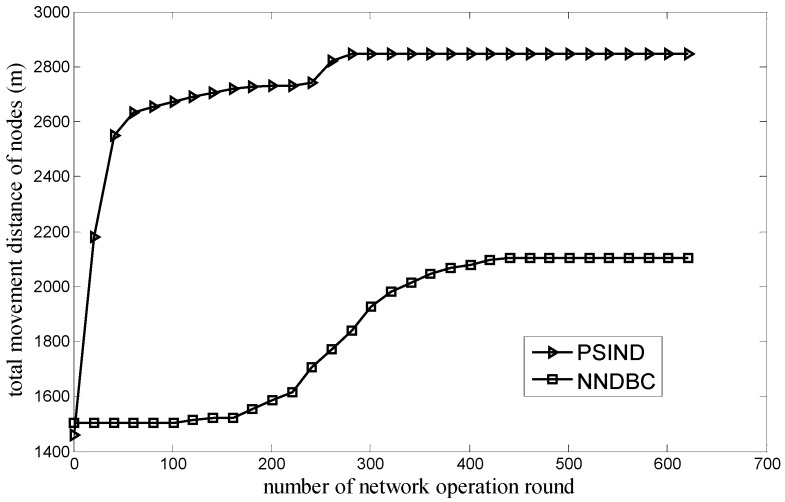
Comparison of total movement distance of nodes during network operation.

Figure 6 shows the comparison of the relationship between the total movement distance of nodes and the round of network operation for the NNDBC and PSIND algorithms, where the number of nodes and events of the simulated scenario are 30 and 100 respectively. Figure 6 shows that if the round of network operation is the same with that of the PSIND algorithm, the NNDBC algorithm can decrease the total movement distance of nodes dramatically. The reason is that during the network operation, the PSIND algorithm has disadvantages that numerous nodes have to move, and nodes may move blindly. However, for the NNDBC algorithm, it defines the concept of aggregate contribution degree along with clustering the nodes, and the nodes with lower aggregate contribution degrees are used to substitute the dying nodes. This is helpful in decreasing the number of nodes that participate in the movement for the network topology adjustment during the network operation and eliminating the blind movement of nodes, thus decreasing the total movement distance of nodes during the network operation.

**Figure 7:**
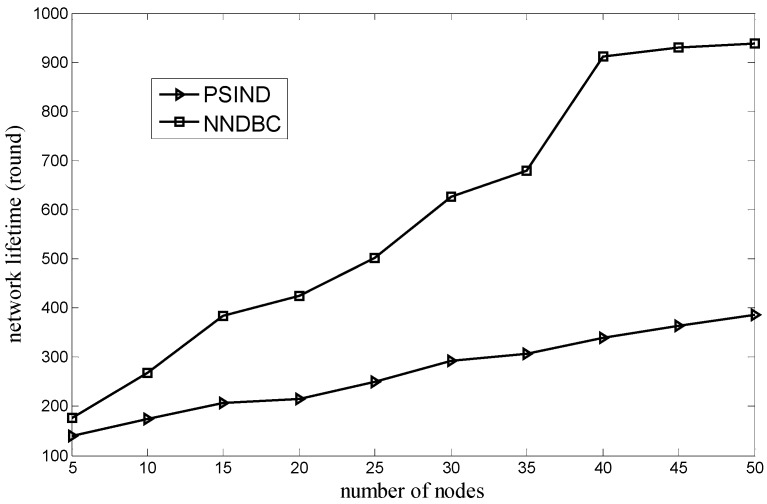
Comparison of network lifetime when number of nodes varies.

**Figure 8:**
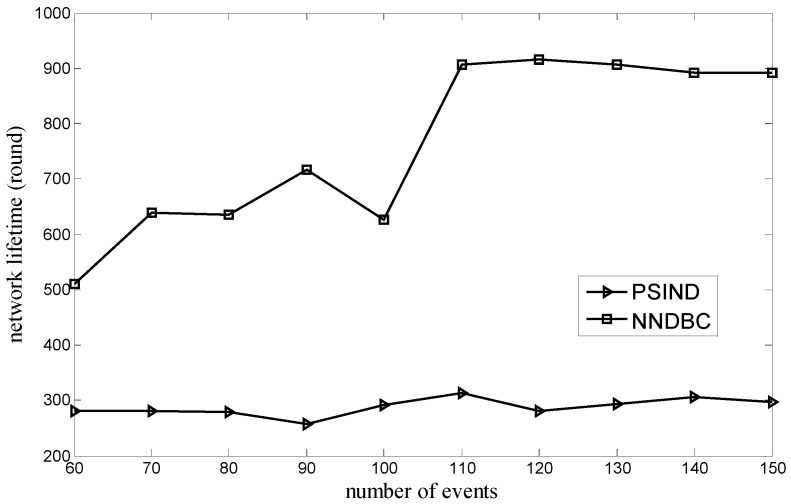
Comparison of network lifetime when number of events varies.

Figure 7 shows the comparison of the relationship between the network lifetime and the number of nodes for the NNDBC and PSIND algorithms, where the number of nodes and the number of events of the simulated scenario are varied and 100, respectively. Figure 8 shows the comparison of the relationship between the network lifetime and the number of events for the NNDBC and PSIND algorithms, where the number of events and the number of nodes of the simulated scenario are varied and 30, respectively. As shown in these figures, if the number of nodes or events is the same as the PSIND algorithm, the NNDBC algorithm can prolong the network lifetime dramatically. The result is attributed to the limited energy of the node and its difficulty to be recharged, as well as the large movement energy consumption in the water. Figure 6 shows the evidence that the total movement distance of nodes of the NNDBC algorithm is much smaller than that of the PSIND algorithm. Therefore, the NNDBC algorithm can decrease the total movement distance of nodes, rendering nodes reserve more energy to sustain longer detection time and prolonging the network lifetime.

## 5. Conclusions

An NNDBC algorithm was proposed for the first time because optimizing network connectivity rate and network lifetime was difficult for the existing node non-uniform deployment algorithms under the premise of improving the network coverage rate for UWSNs. Based on node clustering, the heterogeneous communication ranges of nodes were determined to improve the network connectivity rate, instead of the traditional homogeneous communication ranges. Moreover, the concept of aggregate contribution degree was defined, and the nodes with lower aggregate contribution degrees were used to substitute the dying nodes. Given that this phenomenon can help decrease the number of moving nodes during the deployment and avoid the blind movement of nodes, the total movement distance of nodes was decreased, and the network lifetime was prolonged. Simulation results showed that compared with the typical node non-uniform deployment algorithms for UWSNs, *i.e.*, the PSIND algorithm, the proposed NNDBC algorithm can achieve a better network coverage rate and network connectivity rate, as well as decrease the total movement distance of nodes and prolong the network lifetime. In a real underwater environment, nodes may drift because of the water current; therefore, how to solve the node non-uniform deployment problem consisting of node drift is one of our future study directions. Moreover, establishing a more realistic and exact model to describe node movement energy consumption also deserves our future research.
